# Distinct patterns of socio‐economic disparities in child‐to‐adolescent BMI trajectories across UK ethnic groups: A prospective longitudinal study

**DOI:** 10.1111/ijpo.12598

**Published:** 2019-12-23

**Authors:** Yi Lu, Anna Pearce, Leah Li

**Affiliations:** ^1^ Great Ormond Street Institute of Child Health University College London London UK; ^2^ MRC/CSO Social and Public Health Sciences Unit University of Glasgow Glasgow UK

**Keywords:** BMI, childhood, ethnicity, socio‐economic, United Kingdom

## Abstract

**Background:**

In many high‐income countries, body mass index (BMI)/obesity levels are inversely associated with socio‐economic position (SEP). Little is known whether socio‐economic patterns in BMI trajectories throughout childhood differ by ethnicity, especially in the United Kingdom.

**Objectives:**

To investigate socio‐economic disparities in child‐to‐adolescent BMI trajectories and risks of overweight and obesity during adolescence across ethnic groups.

**Methods:**

Mixed‐effects fractional polynomial and multinomial regression models were applied to estimate socio‐economic differences in BMI trajectories (3‐14 years) and risk of overweight/obesity at 14 years, respectively, in the UK Millennium Cohort Study (n = 15 996). Analysis was stratified by ethnicity.

**Result:**

Poverty was associated with higher BMI in children of White and South Asian origins, with a small difference at 3 years, which widened with age to 0.75 kg/m^2^ (95% CI, 0.59‐0.91) and 0.77 kg/m^2^ (0.26‐1.27) at 14 years for the White and South Asian groups, respectively. There was a reverse income‐BMI association in children of Black (African‐Caribbean) origin with the poverty group having a lower BMI (−0.37 kg/m^2^ [−0.71 to ‐0.04] at 5 years; −0.95 kg/m^2^ [−1.79 to −0.11] at 14 years). These patterns also presented with maternal education as a SEP indicator and for obesity at 14 years.

**Conclusions:**

Socio‐economic advantage may not be universally associated with lower BMI, which should be considered when planning obesity interventions. The positive SEP‐BMI association in children of Black origin requires replication and merits further investigation into underpinning mechanisms.

**Abbreviations**
BMI: Body Mass Index
CI: Confidence Interval
IOTF: International Obesity Task Force
OECD: Organisation for EconomicCo‐operation and Development
RRR: Relative Risk Ratio
SD: Standard Deviation
SEP: Socio‐economic Position
UK: United Kingdom
WHO: World Health Organisation

## 1 INTRODUCTION

Obesity is a global public health challenge.^1^ In many higher‐income countries, levels of body mass index (BMI)^2^, ^3^ and obesity^4^ are socially patterned, with disadvantaged population groups having higher mean BMI and more likely to be affected by obesity, possibly because of their disproportionally greater exposure to risk factors such as consumption of energy‐dense foods.^5^ Recent evidence also suggests that socio‐economic differences in BMI have widened across generations and are emerging at younger ages.^6^, ^7^ High BMI in childhood tends to track into adulthood, and adult obesity is associated with a number of health outcomes,^8^ especially cardiometabolic diseases.

Recent research suggests that the association between socio‐economic position (SEP) and BMI in children may differ by ethnic group. Studies, mainly from the United States, showed that the inverse SEP‐BMI association in children and adolescents is less strong for Asian American populations and inconsistent for Hispanic and non‐Hispanic Black populations, compared with non‐Hispanic White populations.^9^, ^10^, ^11^ The differential associations may be attributable to that cultural, environmental and biological factors related to obesity development have different socio‐economic patterns across ethnic groups.^12^ Little evidence is available in in children in the United Kingdom (UK). One study used data from the National Child Measurement Programme and found that the variation in BMI by area deprivation group is smaller in the South Asian and Black groups than in the White group in London.^13^ A recent cross‐sectional analysis of the UK Millennium Cohort Study (MCS)^14^ showed the relationship between low SEP and increased risk of overweight/obesity at 7 years in White children reversed in Black African/Caribbean children. Examination of these patterns throughout childhood will not only lend greater support to these findings (if replicated) but also point towards when (and thus, potentially, why) these differences occur and whether they persist into adolescence.

Minority ethnic populations represent a growing group in the United Kingdom and are projected to make up a fifth of the population by 2051.^15^ It is therefore necessary to gain a better understanding of how socio‐economic disadvantage impacts on adiposity at different stages in childhood, rather than at one age, across different ethnic groups to provide information for public health policies and targeted interventions. Using longitudinal data from a UK national prospective cohort study, we aimed to investigate (a) socio‐economic disparities in child‐to‐adolescent BMI trajectories across ethnic groups and (b) whether similar patterns of socio‐economic disparities were also found for risks of overweight and obesity during adolescence.

## 2 METHODS

### 2.1 Subjects

The MCS is a nationally representative cohort study, which included over 19 000 children who were born between September 2000 and January 2002 and were living in the United Kingdom at the age of 9 months. The first contact was carried out when participants were 9 months old (baseline). They were followed up at ages of 3, 5, 7, 11, and 14 years. Ethics approval as well as informed consent in writing from parents (and from participants themselves as they were getting older) were obtained.^16^ The MCS oversampled children who lived in the less advantaged socio‐economic circumstances and in England those from minority ethnic backgrounds. Details of the study design are described elsewhere.^17^ We included singletons from White, South Asian, and Black African‐Caribbean backgrounds with at least one BMI measurement at follow‐up visits. Participants whose ethnicity was “mixed” (n = 510, 3.0%), “others” (eg, Chinese) (*n* = 297, 1.8%), or missing (n = 11, 0.07%) were excluded, resulting 16 082 children available for this analysis (eligible sample). After removing participants who had missing data on exposure variables (n = 86), a total of 15 996 participants (99% of eligible sample) with 62 051 BMI measurements were included in the study sample.

### 2.2 Exposure: family SEP indicators

Information on family income was collected at baseline during parental interview and weighted using Organisation for Economic Co‐operation and Development (OECD) scales to take into account family size.^16^ Poverty was defined as OECD equivalized family income below 60% of national median household income—a commonly used measure of relative poverty.^18^ At baseline interviewers, mothers were asked to self‐identify their highest academic qualification from the list—“higher degree,” “first degree,” “diploma in higher education,” “A/AS/S levels,” “O level/GCSE grades A‐C,” “GCSE grades D‐G,” “other academic qualifications (including overseas),” and “none of these qualifications.” Maternal education level was categorized as “higher (GCSE grades A*‐C & above),” “lower (GCSE grades D‐G & below),” and “others.” General Certificate of Secondary Education (GCSE) is a subject‐specific qualification in the United Kingdom typically taken by students at ages 14 to 16 years.

### 2.3 Outcomes: BMI between 3 and 14 years

Height and weight were measured with light clothing and without shoes by trained interviewers at follow‐up visits when children were 3, 5, 7, 11, and 14 years old.^19^ Height was measured to the nearest 0.1 cm using Leicester Stadiometers with heels to the back of the base plate and head in the Frankfurt Plane position. Weight measurements were measured to the nearest 0.1 kg by Tanita BF‐522 W scales. BMI (kg/m^2^) at each age was derived. Weight status at 14 years was categorized as “normal” (including thinness), “overweight” (not including those with obesity), and “obesity” using International Obesity Task Force (IOTF) BMI‐for‐age cut‐offs,^20^ and alternatively World Health Organisation (WHO) BMI‐for‐age cut‐offs.^21^

### 2.4 Ethnicity

We considered ethnicity to be a potential effect modifier of the relationship between poverty and childhood BMI. Participants' ethnicity was defined by parents at baseline using the 2001 UK Census ethnicity classes and subsequently grouped as “White,” “South Asian (Indian, Bangladeshi and Pakistani),” and “Black African‐Caribbean (Black African and Black Caribbean)”.

### 2.5 Statistical analyses

Mixed effects fractional polynomial models were applied to capture the non‐linear trends of BMI changes with age between 3 and 14 years. We considered both second‐order and third‐order fractional polynomials. The third‐order fractional polynomials fitted the data better (Table S1), and the best‐fitting powers were *age*^2^, *age*^2^ * log(*age*), and *age*^3^. Mixed effects models were used to take into account correlations of BMI measurements within individuals and permit the inclusion of cases with missing BMI measurement at some ages under a missing at random assumption.^22^ Random effects were used for *age*^2^ and *age*^3^. Unstructured covariance matrix for the random coefficients was used. Inclusion of an additional random effect for age term (ie, *age*^2^ * log(*age*)) led to nonconvergence. The assessment of model residuals is provided in Table S2. Model selection was guided by deviance, Akaike and Bayesian Information Criterion statistics.

The first model included sex, fractional polynomial age terms, poverty, and the interaction between poverty and each age term. To assess whether the relationship between income poverty and BMI differed by ethnic group, we tested the interaction between ethnicity and poverty. As the *P* value for ethnicity‐poverty interaction was less than.01, the analysis was stratified by ethnicity (ie, the model was repeated for each ethnic group separately).

We also applied multinomial logistic regression models to examine the association between income poverty and overweight/obesity at 14 years. We estimated relative risk ratios (RRRs) and 95% confidence intervals (CIs) for overweight or obesity (vs normal weight) in the poverty group, compared with the nonpoverty group. The models included age at measurement and sex. In addition, to assess whether BMI disparities differed across the BMI distribution, we applied quantile regressions to BMI at 14 years to estimate differences in BMI centiles (ie, 10th, 25th, 50th, 75th, and 90th) between poverty and nonpoverty group by ethnicity, adjusting for sex and age at measurement. We examined whether the relationship between income and BMI was non‐linear by repeating quantile regressions using the continuous income variable and its quadratic term (instead of the binary poverty variable). There was little evidence of nonlinear associations in all ethnic groups (*P* value for quadratic term = 0.01 in White, 0.73 in South Asian, and 0.14 in Black African‐Caribbean). Both multinomial logistic regression and quantile regression models were weighted to take into account clustered sampling design and attrition at 14‐year visits.

### 2.6 Sensitivity analyses

We conducted several additional analyses. First, we repeated mixed effects models using the mother's highest educational level as an alternative SEP indicator. Since the size of the “others” maternal education group was small, for plotting purpose, we estimated BMI differences between “higher” and “lower” maternal education groups. Second, we repeated mixed effects models for boys and girls separately to compare socio‐economic disparities between sexes. BMI is positively correlated with height in children and adolescents.^23^ In our study, children from the nonpoverty group were found to be taller than those from the poverty group in all ethnic groups. Therefore, we additionally adjusted for height in the mixed effects model to examine whether some of the socio‐economic differences in BMI was explained by socio‐economic differences in height.

Analyses were conducted in Stata V.15.1 (Stata Corp., College Station, Texas) and the R software environment for statistical computing V3.6.1.^24^ The following Stata commands were used in this analysis: *fp* for fitting optimal fractional polynomial functions, *mixed* for fitting mixed effects models, and *svy: mlogit* for weighted multinomial logistic regression. The R packages *survey* 25 and *quantreg* 26 were used to fit weighted quantile regression and estimate bootstrap variances.

## 3 RESULTS

### 3.1 Participant characteristics by socio‐economic groups

Of the included 15 996 children, 86.5% were from White, 10.0% from South Asian, and 3.5% from Black African‐Caribbean ethnic backgrounds (Table 1). The percentage of children living in families with relative poverty was higher in the South Asian (64%) and Black African‐Caribbean (59%) groups compared with the White group (32%). Mothers of ethnic minorities were more likely to be in the “lower (GCSE grades D‐G & below)” educational group than those of White ethnic group (South Asian 47.6%, Black African‐Caribbean 39% vs White 26.8%).

**Table 1:** Participants' characteristics by poverty at baseline (total n = 15 996)

	White (n = 13 833)	South Asian (n = 1599)	Black African‐Caribbean (n = 564)	*P* a
	n (%)	n (%)	n (%)	
Sex				.613
Boys	7078 (51.2%)	809 (50.6%)	299 (53.0%)	
Girls	6755 (48.8%)	790 (49.4%)	265 (47.0%)	
Poverty				<.001
Yes	9468 (31.6%)	568 (64.5%)	229 (59.4%)	
No	4365 (68.5%)	1031 (35.5%)	335 (40.6%)	
Maternal education				<.001
Higher (GCSE grades A*‐C & above)	9927 (71.8%)	648 (40.5%)	301 (53.4%)	
Lower (GCSE grades D‐G & below)	3707 (26.8%)	761 (47.6%)	220 (39.0%)	
Others (including gained overseas)	199 (1.4%)	190 (11.9%)	43 (7.6%)	

a Chi‐squared tests for ethnic differences in participants' characteristics.

### 3.2 Socio‐economic inequalities in BMI trajectories according to ethnicity

**Figure 1:**
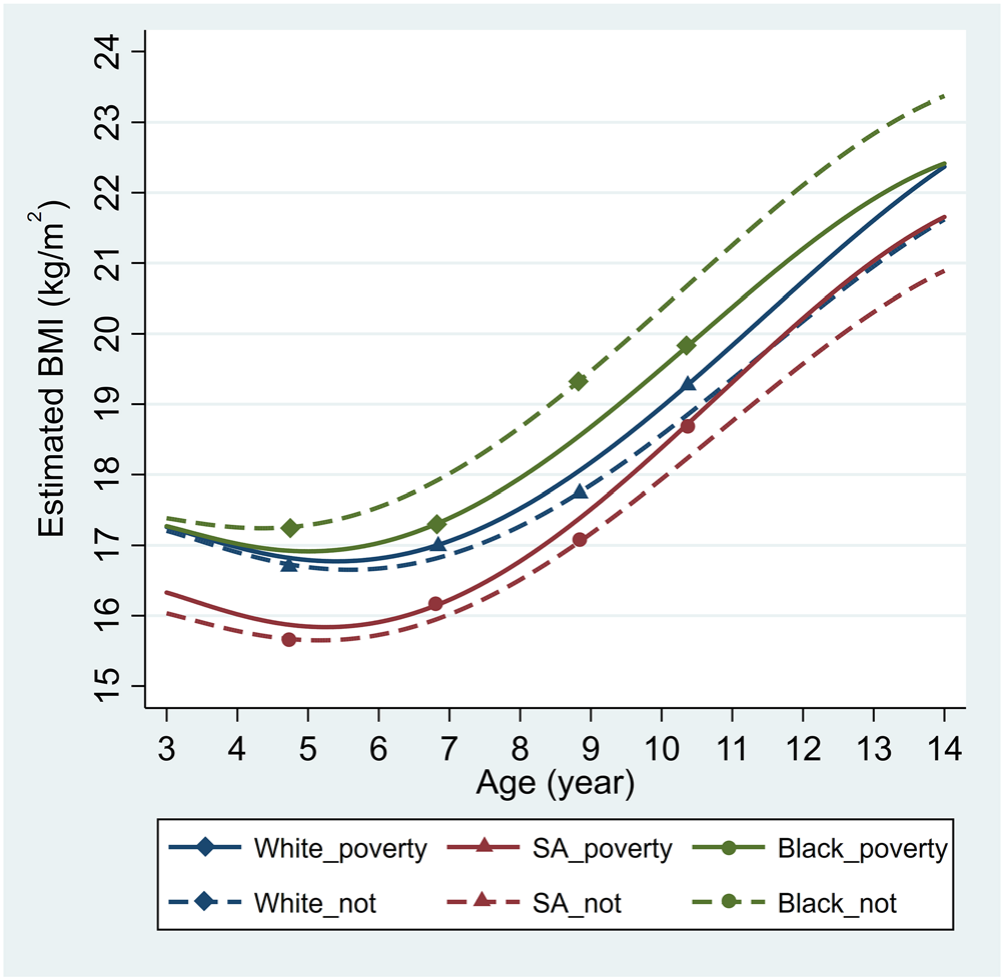
Estimated body mass index (BMI) trajectories between 3 and 14 years by income poverty group, stratified by ethnicity**.** Estimates were based on mixed effects fractional polynomial models, adjusting for sex. The solid (—) lines represent poverty groups while the dashed (– –) lines represent nonpoverty groups. Black: Black African‐Caribbean; not: not in poverty; SA: South Asian

**Figure 2:**
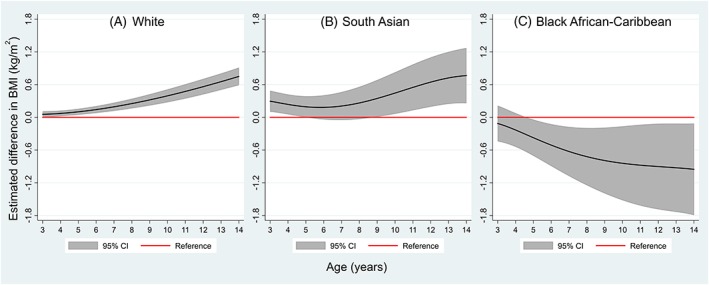
Estimated mean body mass index (BMI) difference and 95% confidence intervals (95% CI) between income poverty and non‐poverty (reference) groups. Models were stratified by ethnic group and included sex, poverty, age terms, and poverty‐age interactions

Overall, children of South Asian ethnic origin had the lowest mean BMI compared with those of White and Black African‐Caribbean origins. Trajectories of mean BMI differed between poverty and nonpoverty groups, and the patterns of these socio‐economic disparities varied by ethnicity (Figure 1). In the White group, a BMI difference between poverty and nonpoverty groups was established at 3 years by 0.05 kg/m^2^ (95% CI, 0.00‐0.11) and increased with age to 0.75 kg/m^2^ (0.59‐0.91) at 14 years (Figure 2A). At 3 years, children of South Asian origin in the poverty group had a higher mean BMI by 0.30 kg/m^2^ (0.10‐0.49) than their counterparts in the nonpoverty group. This difference persisted throughout childhood and widened during adolescence (Figure 2B). However, the association between family income and BMI was the opposite in children of Black African‐Caribbean origin—the poverty group had a lower mean BMI than the nonpoverty group (Figure 2C). The BMI difference emerged at 5 years by −0.37 kg/m^2^ (−0.71 to 0.04) and widened to −0.95 kg/m^2^ (−1.79 to −0.11) at 14 years. The CIs of estimates in the South Asian and Black groups were noticeably wider than those in the White group, owing to their smaller sample sizes. Estimated BMI differences at each age with 95% CIs for each ethnic group are provided in Table S3.

### 3.3 Overweight and obesity at 14 years

Overall, the prevalence of overweight and obesity was 31% in the poverty group and 24% in the nonpoverty group (35% and 29%, respectively, when using WHO references). Table 2 shows estimated RRR for overweight and obesity at 14 years by poverty group and ethnicity. Patterns of income differences in levels of overweight and obesity across ethnic groups were similar to those found for BMI trajectories. Among children of White and South Asian origins, those in the poverty group were more likely to be affected by obesity than their counterparts in the nonpoverty group (RRR = 2.0, 1.6‐2.5 for White; 1.8, 1.1‐3.0 for South Asian). Similar patterns were also seen for overweight, although the RRRs were smaller (RRR = 1.2, 1.0‐1.4 for White) and in the South Asian group with a wide CI (RRR = 1.4, 0.9‐2.3). In children of Black African‐Caribbean origin, poverty was associated with lower relative risk of obesity (RRR = 0.3, 0.1‐0.6), with no evidence of a difference in the relative risk of overweight (RRR = 1.1, 0.7‐1.9).

**Table 2:** Relative risk ratio of overweight and obesity at 14 years by income poverty across ethnic groups, from multinomial logistic regression^a^

		Poverty vs. non‐poverty group	
	Weighted n	RRR	95% CI
White	8968		
Normal		Ref	‐‐
Overweight^b^		1.2	(1.0‐1.4)
Obesity		2.0	(1.6‐2.5)
South Asian	746		
Normal		Ref	‐‐
Overweight^b^		1.4	(0.9‐2.3)
Obesity		1.8	(1.1‐3.0)
Black African‐Caribbean	405		
Normal		Ref	‐‐
Overweight^b^		1.1	(0.7‐1.9)
Obesity		0.3	(0.1‐0.6)

Abbreviation: CI, confidence interval; Ref, reference group; RRR, relative risk ratio.

a Estimated RRR from weighted multinomial logistic regression models. Relative risk ratio (RRR) indicates how the risk ratio (risk of BMI falling in the comparison group vs in the “normal” group) changes with poverty variable. Model was adjusted for age at measurement and sex.

b Overweight group does not include those with obesity.

**Figure 3:**
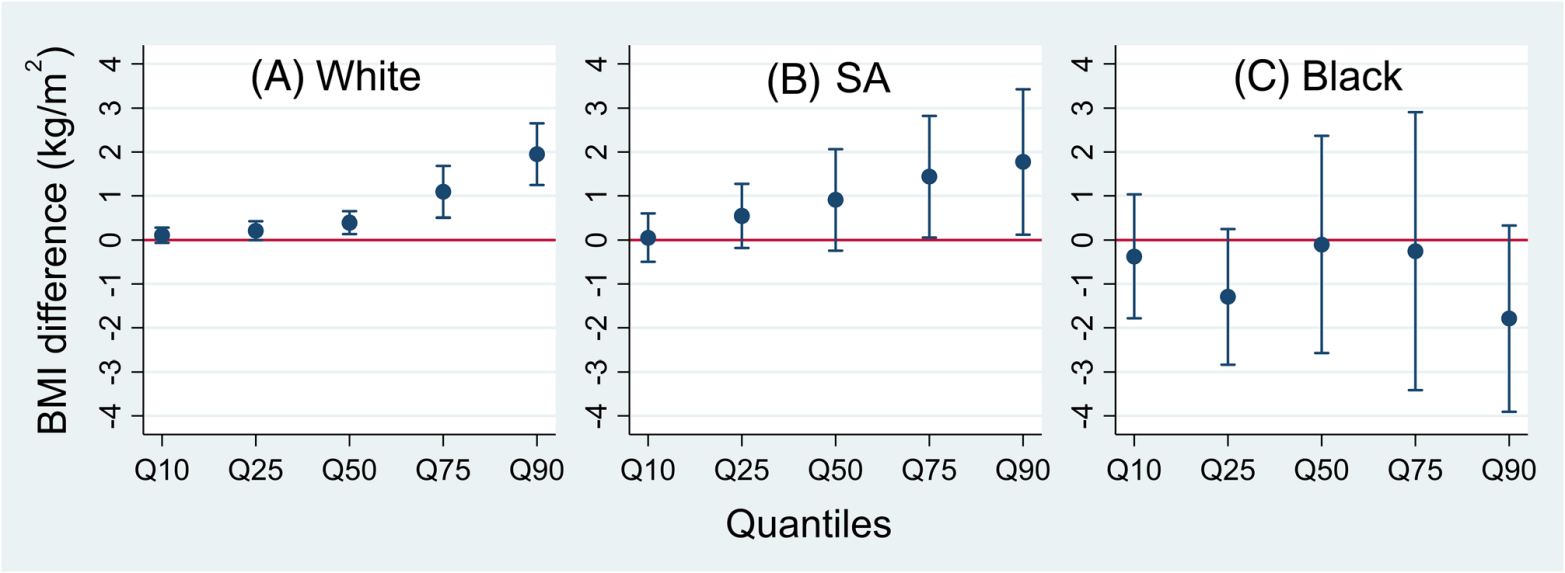
Estimated differences in body mass index (BMI) at 14 years with 95% confidence intervals between poverty and nonpoverty groups at different BMI quantiles. Black: Black African‐Caribbean; SA: South Asian. Data are quantile regression estimates at 10th, 25th, 50th, 75th, and 90th percentiles of BMI distribution. The estimate for the 50th quantile shows the median difference in BMI between poverty and nonpoverty groups. Models were adjusted for sex and age at measurement, with nonpoverty group as the reference group. Error bars are 95% confidence intervals

Quantile regression analyses showed that poverty group had higher BMI than nonpoverty group in children of White and South Asian origins, and the size of BMI difference was greater at higher percentile of BMI distribution (Figure 3). For example, among children of White origin, the BMI difference between poverty and nonpoverty groups was 0.4 kg/m^2^ (0.1‐0.7) at the 50th percentile but greater at the 90th percentile by 2.0 kg/m^2^ (1.3‐2.7). The respective differences in the South Asian group were 0.9 kg/m^2^ (−0.2 to 2.1) and 1.8 kg/m^2^ (0.1‐3.4). Among children of Black African‐Caribbean origin, the poverty group had lower BMI at the 90th percentile (−1.8 kg/m^2^ [−3.9‐0.33]); the pattern of BMI disparities at different percentiles of BMI distribution was less clear. This is consistent with findings on risk of overweight and obesity—poverty was associated with lower risk of obesity but not overweight in the Black African‐Caribbean.

### 3.4 Sensitivity analyses

Socio‐economic patterns in BMI trajectories across ethnic groups largely remained when using maternal education level as an alternative family socio‐economic indicator. However, the increment in estimated BMI differences with age was smaller, and the standard errors of the estimates were greater for minority ethnic groups (Figure S1). Analysis further stratified by sex revealed the same socio‐economic patterns for boys and girls. Estimated BMI differences between poverty groups were slightly greater in girls than in boys for White and South Asian groups but were similar between boys and girls in the Black African‐Caribbean group (Figure S2). However, the 95% CIs of these estimates for boys and girls overlapped across all ages. The 95% CIs were markedly wider compared with results from the analysis with both sexes combined, possibly because of smaller sample sizes. Adjusting for height had little effects on estimated BMI differences (data not shown).

## 4 DISCUSSION

In this contemporary UK national cohort, our main findings include (a) child‐to‐adolescent BMI trajectories were socio‐economically patterned, and the pattern differed between ethnic groups. Income deprivation was associated with higher BMI in the White and South Asian groups but with lower BMI in the Black African‐Caribbean group. (b) A difference in BMI between poverty and nonpoverty groups was established as early as 3 years in the White and South Asian groups, and overall increased with age across all ethnic groups. (c) Similar socio‐economic patterns presented when using maternal education as the alternative SEP indicator in sensitivity analyses and were found for the risk of obesity at 14 years.

Our findings on socio‐economic disparities in BMI in the White and South Asian ethnic groups are consistent with existing evidence from the general population in the United Kingdom. We showed that a modest difference in mean BMI between poverty and nonpoverty groups was established at as early as 3 years and increased with age. This is in line with previous studies, which suggested that social differences in BMI emerged at younger ages in the general population^6^, ^7^ and widened with age.^27^ One UK study (approximately 96% participants from White ethnic background) found that a socio‐economic difference in BMI presented at about 4 years and became greater with age (0.4 kg/m^2^ for boys and 0.9 kg/m^2^ for girls at 10 years).^27^ The differences in factors such as dietary between social groups from a young age may have are likely to largely explain these BMI differences. However, as detailed dietary measures were not available in the MCS, we were not able to fully investigate this. In addition, we found, as suggested by previous studies,^6^, ^28^ that BMI disparities at 14 years were greater at higher end of the BMI distribution in the White and South Asian groups.

Limited studies have investigated socio‐economic disparities in BMI trajectories in UK children across ethnic groups. Findings from US studies are mixed—some studies showed a negative^29^, ^30^ or no association^9^, ^31^, ^32^ between SEP and BMI/obesity for non‐Hispanic Black children, other studies reported a positive association as shown in our study.^10^, ^33^ A recent analysis of the MCS at age 7 showed that White children in lower income families (bottom 40%) were at increased risk of overweight/obesity at 7 years, compared with their counterparts in higher‐income (top 60%) families; the relationship was reversed for Black African/Caribbean children.^14^ Our study supports these findings and, in addition, demonstrates that these differences emerged at around age 6 years and persisted to 14 years. Similar patterns were also seen for obesity at 14 years. BMI in children and adolescents is positively correlated with height.^23^ Although mean height of Black African‐Caribbean children in the nonpoverty group was greater than that of their counterparts in the poverty group, the socio‐economic pattern in BMI was not explained by socio‐economic difference in height (data not shown). The underlying mechanisms of this socio‐economic pattern are unclear. It is likely that socio‐economic factors interact with cultural and dietary factors differently across ethnic groups and therefore influence BMI/obesity development.^12^ There is some evidence from qualitative studies that perceptions of desirable or healthy body size differ between different cultures.^34^ US studies showed that greater acculturation after immigration was associated with higher income but with lower vegetable consumption^35^ and other unhealthier dietary practices.^36^ Similar findings were also reported in the United Kingdom. Maternal health behaviours worsen after moving to the United Kingdom and with increasing duration of residence in the United Kingdom.^37^ It may be that families from less advantaged background in our sample were less acculturated than those from more advantaged families and thus less vulnerable to the unhealthy elements of western diets and more protected by their traditional diets. This is supported by a recent systematic literature review.^38^ Nevertheless, we were not able to examine why this socio‐economic pattern in BMI was only observed in the Black African‐Caribbean group but not in the South Asian group. Future studies are warranted to provide more insights into how immigration, acculturation, and socio‐economic disadvantage interacts with BMI across different ethnic groups.

BMI disparities between poverty and non‐poverty groups among children of White and South Asian origins were established as early as 3 years in our study and overall widened with age. High BMI in childhood and adolescence tends to track into adulthood, and adult obesity is related to a number of health outcomes,^8^ eg, cardiometabolic health. Howe demonstrated that by the age of 10 years, there were not only marked BMI differences between the highest and lowest maternal education groups but also evidence of social disparities in several cardiovascular risk markers.^39^ There is, therefore, an urgent need for early intervention to reduce socio‐economic inequalities in BMI. The distinct socio‐economic patterns in BMI between ethnic groups shown in our study indicate that higher SEP may not be universally associated with lower BMI. Therefore, public health approaches to promote healthy weight need to consider the varying needs of their target populations. Interventions and programmes addressing socio‐economic disadvantage will benefit the health and well‐being of these families in many ways, but other approaches may be needed to reduce the higher rates of BMI/obesity observed among children of Black African‐Caribbean origin.

Our analysis benefited from using family‐level socio‐economic indicators collected during early childhood, which have been suggested to be more accurate than those retrospectively collected.^40^

We used repeated measures of BMI, which allowed us to explore the age at which socio‐economic differences in BMI emerge and how they change with age. However, a few limitations need to be noted. Despite using a large national study, which oversampled minority ethnic groups, the sample sizes of South Asian and Black African‐Caribbean groups are relatively small, which contributed to the wider confidence intervals of estimates in these groups, especially in quantile regression analyses and when analyses were further stratified by sex. Previous research has shown that BMI differences among relatively heavy children are largely driven by differences in fat mass.^41^ A social gradient was observed in children's fat mass but not in lean mass or trunk fat mass.^42^ BMI does not distinguish between fat mass and lean mass,^23^ and there are known ethnic differences in body composition in UK children with those of Black African‐Caribbean origin having a lower level of body fat at a given BMI compared with those of White and South Asian origins.^43^ The present study was not able to ascertain whether the socio‐economic pattern in BMI in the Black African‐Caribbean group was primarily attributed to differences in lean mass or fat mass. Mothers of minority ethnic groups were more likely to self‐report their education level in the “others” group, which potentially captured a range of different qualifications obtained overseas. Therefore, the estimated BMI differences between lower and higher maternal education groups among children of ethnic minorities in the sensitivity analysis may be underestimated. Detailed information on dietary intakes from a young age was not collected in the MCS, which prevents further investigation on whether social patterns in diet also differed across ethnic groups.

## 5 CONCLUSIONS

Socio‐economic disadvantage may not be universally associated with higher child and adolescent BMI. Among children of Black African‐Caribbean origin, we found that poverty was associated with lower BMI. Socio‐economic disparities in BMI emerged (in the expected direction) as early as 3 years in White and South Asian groups and generally increased with age throughout childhood into adolescence, highlighting the need of early intervention to reduce socio‐economic disparities in BMI. The reverse socio‐economic gradient among children of Black African‐Caribbean origin requires replication but indicates that public health approaches to promote healthy weight need to consider the varying needs of their target populations. Future studies are needed to fully understand the mechanisms underpinning the influence socio‐economic factors on BMI in different ethnic groups.

## CONFLICT OF INTEREST

The authors have no conflicts of interest relevant to this article to disclose.

## AUTHOR CONTRIBUTIONS

Y.L. and L.L. conceptualized the study. Y.L. conducted the analyses, interpreted the results, and drafted the initial manuscript. All authors contributed to the design of the study and interpretation of the data, critically revised the manuscript, and approved the submitted manuscript.

## ETHICS STATEMENT

The MCS has obtained ethics approval for each of its surveys. Informed consent in writing was obtained from parents, as well as participants themselves as they grew older. All MCS data used in this analysis can be accessed via the UK Data Service (University of Essex and University of Manchester). The persistent identifiers are first survey https://doi.org/10.5255/UKDA-SN-4683-1; second survey http://doi.org/10.5255/UKDA-SN-5350-3; third survey http://doi.org/10.5255/UKDA-SN-5795-3; fourth survey http://doi.org/10.5255/UKDA-SN-6411-6; fifth survey http://doi.org/10.5255/UKDA-SN-7464-2; and sixth survey http://doi.org/10.5255/UKDA-SN-8156-2.

## Supporting information

Table S1: Comparison of deviance between first‐, second‐ and third‐order fractional polynomial models for BMI between 5 and 14 years*
Table S2: Summary of level‐1 residuals (kg/m^2^) from unadjusted mixed effects fractional polynomial model, by age
Table S3: Estimated mean BMI differences (kg/m^2^) with 95% confidence intervals between poverty and non‐poverty groups from mixed effects fractional polynomial models
Figure S1: Estimated mean BMI differences with 95% confidence intervals from 3 to 14 years between lower and higher maternal education groups. Models were adjusted for sex. Higher maternal education group (i.e. GCSE grades A*‐C & above) was the reference group.
**Figure S2: Estimated mean BMI difference and 95% confidence intervals (95% CI) between income poverty and non‐poverty (reference) group, by ethnicity and sex.**
