# Supplementary material for: Distinct patterns of socio‐economic disparities in child‐to‐adolescent BMI trajectories across UK ethnic groups: A prospective longitudinal study
Source: Pediatr Obes. 2019 Dec 23;15(4):e12598. doi: 10.1111/ijpo.12598 (PMC7079192; doi:10.1111/ijpo.12598)
Supplement: Supplementary file 1 — Table S1: Comparison of deviance between first‐, second‐ and third‐order fractional polynomial models for BMI between 5 and 14 years* Table S2: Summary of level‐1 residuals (kg/m2) from unadjusted mixed effects fractional polynomial model, by age Table S3: Estimated mean BMI differences (kg/m2) with 95% confidence intervals between poverty and non‐poverty groups from mixed effects fractional polynomial models Figure S1: Estimated mean BMI differences with 95% confidence intervals from 3 to 14 years between lower and higher maternal education groups. Models were adjusted for sex. Higher maternal education group (i.e. GCSE grades A*‐C & above) was the reference group. Figure S2: Estimated mean BMI difference and 95% confidence intervals (95% CI) between income poverty and non‐poverty (reference) group, by ethnicity and sex. [file IJPO-15-e12598-s001.docx]

Supplementary materials

**Table S1:** **Comparison of deviance between first-, second- and third-order fractional polynomial models for BMI between 5 and 14 years***

| **Model** | **DF** | **Powers** | **Deviance** | **AIC** | **BIC** | **Level-1 residual variance** |
| --- | --- | --- | --- | --- | --- | --- |
| Second-order | 10 | $age, age*\log\left( age \right)$ | 237138 | 237158 | 237248 | 0.918 |
| Third-order | 11 | $age^{2}, age^{2}*\log\left( age \right), age^{3}$ | 231789 | 231811 | 231910 | 0.621 |

* In the second-order model, random effects were allowed for both age terms. In the third-order model, random effects were allowed for $age^{2}$ and $age^{3}$; inclusion of random effects for the additional age term led to non-convergence. DF: degrees of freedom; AIC: Akaike information criterion statistics; BIC: Bayesian information criterion statistics.

Table S2: Summary of level-1 residuals (kg/m^2^) from unadjusted mixed effects fractional polynomial model, by age

| **Age (year)** | **n** | **mean** | **SD** |
| --- | --- | --- | --- |
| 3 | 12,397 | 0.00 | 0.58 |
| 4 | 834 | 0.03 | 0.53 |
| 5 | 11,413 | 0.01 | 0.56 |
| 6 | 2,507 | -0.01 | 0.57 |
| 7 | 10,195 | -0.02 | 0.65 |
| 8 | 2,490 | 0.03 | 0.62 |
| 9 | 11 | 0.12 | 0.58 |
| 10 | 9,521 | 0.02 | 0.55 |
| 11 | 2,455 | -0.05 | 0.53 |
| 12 | 17 | -0.11 | 0.28 |
| 13 | 7,324 | -0.01 | 0.35 |
| 14 | 2,887 | 0.01 | 0.28 |

*n: number of observations available; SD: standard deviation.

**Table S3: Estimated mean BMI differences (kg/m^2^) with 95% confidence intervals between poverty and non-poverty groups from mixed effects fractional polynomial models**

|  | **White** | | **South Asian** | | **Black African-Caribbean** | |
| --- | --- | --- | --- | --- | --- | --- |
| **Age** | **diff*** | **95% CI** | **diff*** | **95% CI** | **diff*** | **95% CI** |
| 3 | 0.05 | (0.00, 0.11) | 0.30 | (0.10, 0.49) | -0.11 | (-0.44, 0.21) |
| 4 | 0.07 | (0.02, 0.12) | 0.24 | (0.05, 0.42) | -0.23 | (-0.54, 0.08) |
| 5 | 0.10 | (0.05, 0.16) | 0.19 | (0.00, 0.39) | -0.37 | (-0.71, -0.04) |
| 6 | 0.14 | (0.08, 0.21) | 0.18 | (-0.04, 0.40) | -0.51 | (-0.89, -0.13) |
| 7 | 0.19 | (0.12, 0.27) | 0.21 | (-0.05, 0.46) | -0.63 | (-1.08, -0.18) |
| 8 | 0.25 | (0.16, 0.34) | 0.26 | (-0.03, 0.56) | -0.72 | (-1.25, -0.20) |
| 9 | 0.32 | (0.22, 0.42) | 0.35 | (0.01, 0.68) | -0.79 | (-1.40, -0.19) |
| 10 | 0.39 | (0.28, 0.51) | 0.44 | (0.06, 0.83) | -0.84 | (-1.52, -0.17) |
| 11 | 0.47 | (0.34, 0.61) | 0.55 | (0.13, 0.97) | -0.88 | (-1.62, -0.14) |
| 12 | 0.56 | (0.42, 0.70) | 0.65 | (0.19, 1.10) | -0.90 | (-1.69, -0.12) |
| 13 | 0.65 | (0.50, 0.80) | 0.72 | (0.25, 1.20) | -0.93 | (-1.73, -0.12) |
| 14 | 0.75 | (0.59, 0.91) | 0.77 | (0.26, 1.27) | -0.95 | (-1.79, -0.11) |

*****estimated mean BMI difference in kg/m^2^. CI: confidence interval. Models included sex, poverty, age terms and poverty-age interactions, stratified by ethnic group.


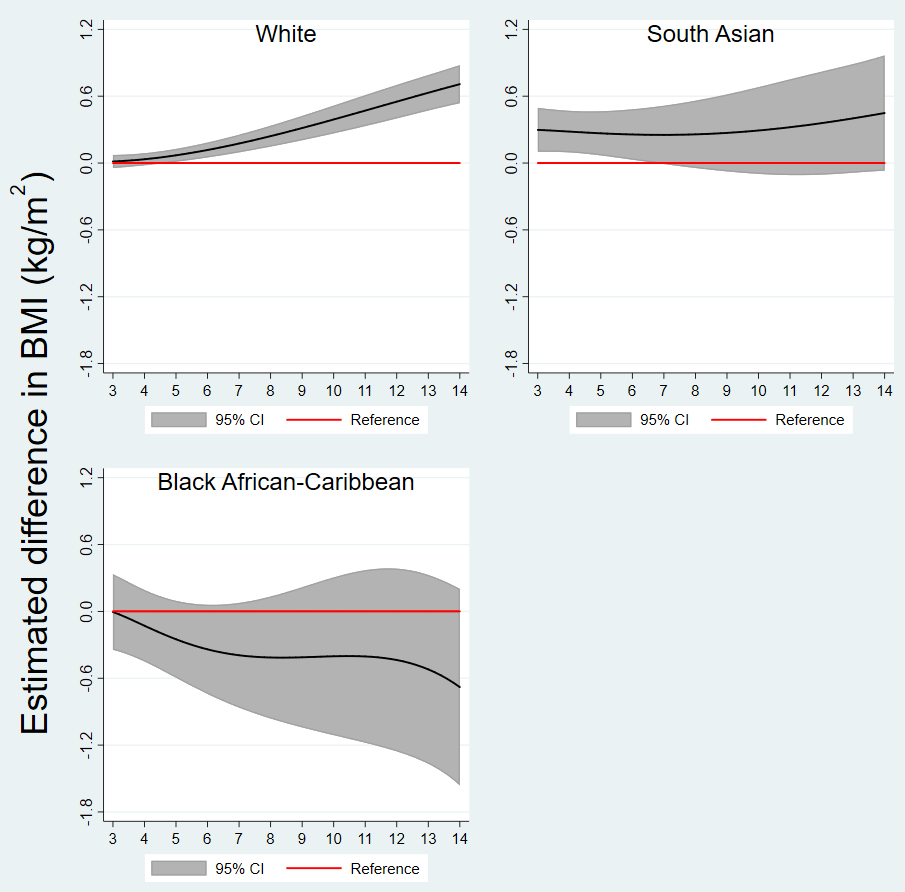


Figure S1: Estimated mean BMI differences with 95% confidence intervals from 3 to 14 years between lower and higher maternal education groups. Models were adjusted for sex. Higher maternal education group (i.e. GCSE grades A*-C & above) was the reference group.


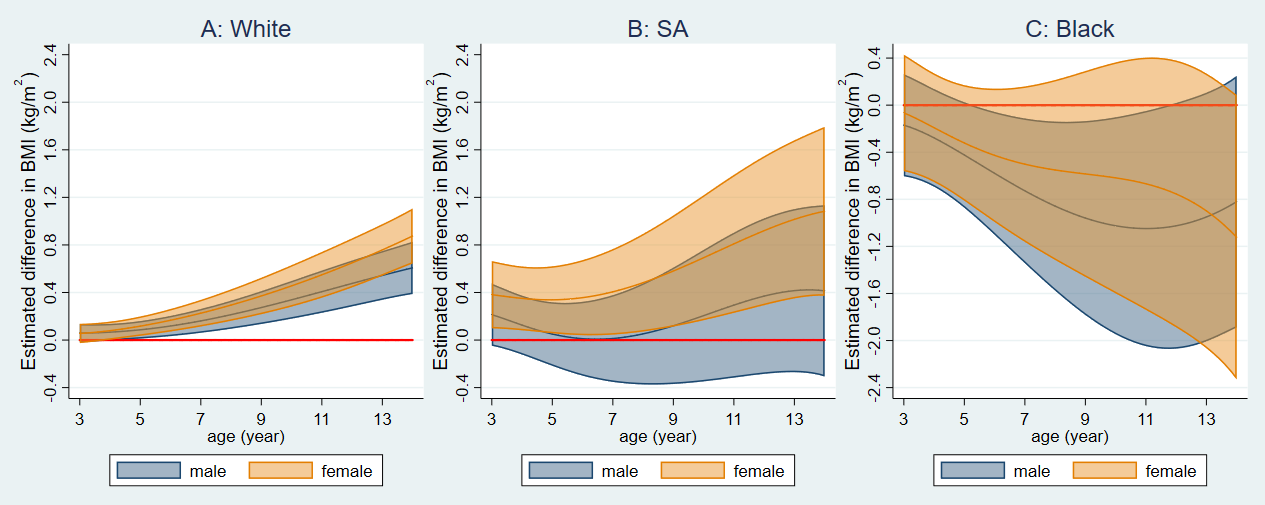


**Figure S2: Estimated mean BMI difference and 95% confidence intervals (95% CI) between income poverty and non-poverty (reference) group, by ethnicity and sex.**

Models included, poverty, age terms and interaction between poverty and all age terms. Models were fitted for each ethnic and sex group separately (i.e. six models were conducted). SA: South Asian; Black: Black African-Caribbean.
